# BRCA1 mutation in the Triple- Negative Breast Cancer Group

**DOI:** 10.1186/1897-4287-10-S4-A15

**Published:** 2012-12-10

**Authors:** Jelena Maksimenko, Arvids Irmejs, Genadijs Trofimovics, Edvins Miklasevics

**Affiliations:** 1 Riga Stradins University, The Institute of Oncology, Latvia

## Introduction

Approximately 75% of BRCA1 mutation-related breast cancers are triple- negative breast cancers (TNBC). Recent studies have proposed that individuals diagnosed with (TNBC) before age 50 should be tested for BRCA1 mutation.

## Aim of the study

To investigate the prevalence of germline BRCA1 founder mutations in patients with TNBC and to find out appropriate indications for BRCA1 mutation testing in patients with TNBC.

## Material and methods

332 unselected patients with invasive breast cancer diagnosed between 2006- 2011 were identified from Pauls Stradins Clinical University Hospital, Riga East University hospital databases and clinical data from medical records were retrospectively analyzed. All patients were tested for the two common founder mutations in BRCA1 (4153delA and 5382insC) in Latvia using a multiplex- specific polymerase chain reaction (PCR) assay.

## Results

Of the 332 patients, 100 (30.1%) were identified as having TNBC, the remaining patients 232 (69.9%) were identified as having other breast cancer subtypes. Among patients with TNBC the BRCA1 mutations prevalence were 17% (17 of 100 cases) compared to other patients’ group with 0.86% (2 of 232 cases) BRCA1 mutation prevalence (P= 0.001).

In the TNBC group BRCA1 mutation carriers were significantly younger at diagnosis than non-carriers (median age, 53.6 years versus 57 years, respectively; P= 0.043). The vast majority of BRCA1 mutations within the TNBC group were diagnosed in women under 50 years of age- in 10 cases (58.8%), in 5 cases (29.4%) BRCA1 mutations were detected between 50 and 65 years of age and in 2 cases (11.8%)- in patients older than age 65 years. In 113 of 332 (34%) patients without family history of cancer 2 of 19 BRCA1 mutations were found, in 219 (66%) of 332 patients with family history of cancer 17 of 19 BRCA1 mutations were detected, but BRCA1 mutation was not statistically significant associated with a family history of cancer and age of breast cancer diagnosis.

## Conclusion

Our study data indicates a high prevalence of BRCA1 mutation in TNBC and suggests BRCA1 mutation testing for all patients with TNBC regardless of family history and age at diagnosis of breast cancer.

